# Self-reported explanations for self-injury by people with intellectual disabilities: a systematic review of qualitative studies

**DOI:** 10.1080/20473869.2022.2098665

**Published:** 2022-07-19

**Authors:** Beverley Samways, Pauline Heslop, Sandra Dowling

**Affiliations:** 1 School for Policy Studies, University of Bristol, Bristol, UK; 2 Professor of Intellectual Disabilities Studies, University of Bristol, Bristol, UK; 3 Senior Lecturer, School, for Policy Studies, University of Bristol, Bristol, UK

**Keywords:** self-injury, self-injurious behaviour, self-harm, intellectual disabilities, systematic review

## Abstract

**Background:**

Emotional distress has received less attention as an explanatory factor for self-injury in people with intellectual disabilities, with research and practice primarily focusing on biobehavioural factors. This systematic review examines the self-reported explanations for self-injury by people with mild or moderate intellectual disabilities, and discusses how the findings contrast with those from self-reported studies of people within the general population who self-harm.

**Methods:**

Five databases (PsychINFO, IBSS, CINAHL, Web of Science and Medline) were systematically searched to find qualitative, empirical research since 2000 about self-reported reasons for self-injury.

**Results:**

Four studies were found which conducted research with people with intellectual disabilities. Three primary themes are discussed: relief from overwhelming emotions; trauma and loss; and difficulty in articulating emotions.

**Conclusion:**

This review found a paucity of research asking people with intellectual disabilities about their own self-injury. However, the research available suggests that explanatory factors for self-injury typically reported in the general population should be considered for those with mild or moderate intellectual disabilities.

## Introduction

‘Self-injurious behaviour’ (SIB) or ‘self-injury’ is often the term used to describe self-harming behaviours presented by people with intellectual disabilities (Heslop and Lovell 2013; NICE 2015).^1^ The National Institute for Clinical Excellence (NICE)^2^ provide comprehensive guidelines for Challenging Behaviour and Learning Disabilities^3^ (2015), including self-injury; these state that self-injury is ‘repeated, self-inflicted behaviour, such as people hitting their head or biting themselves’ (p. 29).

Self-injury is a serious health concern, which is often intransient (Oliver and Richards 2015). Prevalence in people with intellectual disabilities is estimated to be between 4% and 24%, which typically increases with the severity of intellectual disability (Oliver and Richards 2015). Self-injury is a profoundly challenging issue for those with intellectual disabilities, as well as the families and professionals supporting them (Folch *et al.* 2018; Minshawi *et al.* 2015). Alongside the potential for physical harm, self-injury can impact on the development of social and adaptive skills, as well as overall quality of life (Bradley *et al.* 2018; Minshawi *et al.* 2015). Additionally, chronic self-injury can result in more restrictive placements, which are more likely to be outside a person’s community (Chezan *et al.* 2017; Minshawi *et al.* 2015); this potentially isolates a person from family and support networks.

Explanations for self-injury amongst people with intellectual disabilities have predominantly been researched from a biobehavioural perspective, examining biological reasons why a person might hurt themselves or behavioural reasons as to how self-injury is functionally established (Hagopian *et al.* 2018). This has influenced policy and practice in the UK (BILD 2016; NICE 2015) and is echoed in the practices of applied behavioural analysis (Gregori *et al.* 2018; Shkedy *et al.* 2019), which is based on the premise that self-injury becomes functional in achieving particular goals and is reinforced by the response it engenders (Minshawi *et al.* 2015). For example, a child may first self-injure out of frustration or in response to pain, but, finding that an often rapid response follows the behaviour, inadvertently learns that this is the fastest way to engender help; this may be exacerbated by a child having very few functional communication skills to otherwise draw on.

The NICE Guidelines for Challenging Behaviour and Learning Disabilities (2015) state that self-injury may indicate pain, distress or ‘another purpose’, such as communication (p. 29). NICE (2015) also recommend that the assessment of self-injury should include ‘all current and past personal and environmental factors’ (p. 16) and that the areas of self-neglect, breakdown of family or residential support, depression, exploitation, abuse and neglect by others are kept under regular assessment. These adverse experiences, recognised as possible antecedents for self-injury in people with intellectual disabilities, imply that emotional distress is tacitly associated with self-injury, though this explanatory factor has received less attention in research about this population (Jones *et al.* 2004).

Research into self-harm in the general population^4^ is well-established, including research directly involving people who self-harm (Cipriano *et al.* 2017; Nock 2009); most of this body of research considers emotional distress as an explanatory factor (Klonsky 2009; Nock 2009). The inclusion of people with intellectual disabilities in this research has been limited, in part because of the apparent difficulty in including people with intellectual disabilities in research; some of these difficulties include communication barriers and complexities in gaining informed consent (Brown and Beail 2009; Klotz 2004). NICE (2015) state the importance of developing a ‘shared understanding about the function of the behaviour’ (p. 7) and involving people with intellectual disabilities in decisions about care. Thus, it is essential that, despite the challenges, we seek to understand as much as we can about self-reported explanations for self-injury by people with intellectual disabilities.

This aim of the systematic review was to explore what is already known about explanations of self-injury from the perspective of people with intellectual disabilities who are able to self-report; the systematic review synthesises the evidence from empirical studies which explored the explanations people with intellectual disabilities gave for their own self-injury. The review therefore addressed the research question: ‘what reasons do people with intellectual disabilities (who are able to self-report) give for their own self-injury?’ After presenting the findings, we then discuss how they contrast with those from self-reported studies with people within the general population who self-harm^5^ and the wider context of research with people with intellectual disabilities who self-injure.

## Methodology

Five bibliographic databases, identified with the support of a subject librarian, were searched in May 2020:

- PsychINFO (Psychiatry, psychology and social sciences)
- IBSS (International Bibliography of the social sciences)
- CINAHL (Nursing and allied health)
- Web of science (Social sciences, arts and humanities)
- Medline (US National online library of medicine).

Each of the databases was searched using all combinations of the words ‘self-injury’ and ‘cause’. Search terms were developed through looking at relevant systematic review search terms (e.g. Van den Bogaard *et al.* 2019; Griffith *et al.* 2013), and utilising any suggested alternatives in the databases. An example of a database specific search strategy (MEDLINE) is given in Table 1; the same search terms were used across the databases.

**Table 1:** Example search terms

(self-harm or "self harm" or self-injur* or "self injur*" or "self-injurious behav*" or NSSI or "non-suicidal self-harm" or "non-suicidal self-injur*" or self-mutilat* or self-destruct* or "non-suicidal behav*" or "challenging behav*" or "behav* that challenges" or "problem behav*" or "self-inflicted injur*" or self-poisoning or self-cutting)
AND
(cause* or reason* or "contributing factor*" or "explanatory factor*" or explanation* or "causative factor*" or causation* or explorat* or causal* or motiv* or experience* or perception* or perspective* or "personal perspective*" or understand* or reflect* or attribution* or view* or function* or intention*or drive* or purpose*) adj10 (patient* or client* or user* or person* or people*)

Search limits were set to ensure results only included peer-reviewed articles from 2000 onwards. Initial feasibility searching indicated that, whilst developments in understanding self-injury have moved rapidly (McManus *et al.* 2019), research seeking self-reported reasons for self-injury from people with intellectual disabilities was sparse, and a twenty-year span was necessary to capture a reasonable sample of studies. The search was limited to English language articles, reflective of the concern with UK policy; screening also excluded non-empirical studies.

### Inclusion/exclusion criteria

The inclusion/exclusion criteria were first applied at the title and abstract review stage and again when later reading full text articles. Table 2 lists the inclusion and exclusion criteria in full.

**Table 2:** Inclusion and exclusion criteria

**Inclusion criteria**
Peer-reviewed research Published from 2000-present English language full text Empirical research that includes direct citation from participants Qualitative studies—interviews, semi-structured interviews or written text Research exploring the causes of self-harm Self-report studies that ask participants who have direct experience of self-harm
Exclusion criteria
Not peer-reviewed or status unclear (limits set in search where possible) Articles published prior to 2000 (limits set in search where possible) Non-English language articles (limits set in search where possible) The study was not empirical. Majority of the self-report evidence was through non-qualitative methods, i.e. Likert-scales and measures. Studies did not seek causal evidence (e.g. studies that explore why someone stopped self-harming) Studies that asked participants about other people’s self-harm Majority of data was from professionals, families or observation Did not delineate between self-harm and suicidal behaviour, or primarily described suicidal behaviour. Did not delineate between self-harm and challenging behaviour

Some studies sought self-reported reasons for self-injury from people with intellectual disabilities within a wider inquiry into reasons for ‘challenging behaviour’^6^. Whilst these provided some useful evidence, self-injury may, or may not, be one of the behaviours included under the umbrella term ‘challenging behaviour’, and it was not usually possible to disaggregate the behaviours in question. After discussion between the research team, it was agreed to exclude these papers; this is further explored in the discussion. Some studies sought attributional opinions from peers, professionals, families or observational data. Studies were excluded if such data dominated the findings or could not be extricated from the self-report data. Thus, this review focused specifically on qualitative data gathered directly from those with intellectual disabilities who self-injure.

### Risk of bias

Just under half of the excluded articles (six of thirteen) were combined with half of the included articles (two). These eight articles were screened against the exclusion and inclusion criteria by a second reviewer. There was agreement about which articles to include and exclude; discussions between the reviewers resulted in the strengthening of the wording of the exclusion and inclusion criteria.

### Quality assessment

The Critical Appraisal Skills Programme (CASP) (2018) Qualitative Studies Checklist was used to assess the methodological rigour of the studies; this is the most frequently used tool for qualitative studies (Majid and Vanstone, 2018). The checklist poses 10 questions about aims, methodology, design, recruitment, data collection, ethics and reflexivity, analysis, validity and rigour. The questions can be answered ‘yes’, ‘no’ or ‘can’t tell’. Studies scored one for every ‘yes’: thus, 10 indicated a study of the highest quality. Quality assessment was recorded in tabular form, allowing for notes to be made against the decision making. (See Table 3 for an example.)

**Table 3:** Example quality assessment table

Author	Brown, J. & Beail, N.			
**Title**	Self-harm among people with intellectual disabilities living in secure service provision: a qualitative exploration.			
**Date**	2009			
**CASP Checklist**	**Yes**	**Can't Tell**	**No**	**Comments**
**Section A: Are the results valid?**				
1) Was there a clear statement of the aims of the research?	1			Clearly stated in abstract and expounded on p. 505
2) Is a qualitative methodology appropriate?	1			Yes—exploring participants' experiences and understanding
3) Was the research design appropriate to address the aims?	1			Yes—semi-structured interview study
4) Was the recruitment strategy appropriate to the aims of the research?		1		Potential participants identified in a secure unit. It's not clear how the participants were invited to be part of it, or how they understood that they had been chosen.
5) Was the data collected in a way that addressed the research issue?	1			Semi-structured interview schedule, with participants having some control over the direction of the conversation, etc.
6) Has the relationship between the researcher and participants been adequately considered?		1		The relationship is not made clear although implies that there is not a clinical relationship; wider context considered in limitations. The presence of a staff member during interviews is discussed in limitations—p. 511. Some consideration given to location and privacy, p. 505.
Section B: What are the results?				
7) Have ethical issues been taken into consideration?	1			Ethical approval confirmed and some documentation of confidentiality considered.
8) Was data analysis sufficiently rigorous?	1			IPA analysis is explained and justified.
9) Is there a clear statement of findings?	1			Yes. Well themed and illustrated with quotes.
10) How valuable is the research?	1			Very valuable as this research is rare
Total	8	2	0	

The second reviewer quality assessed half of included studies. The scoring was within 10% difference; the areas of disagreement were discussed and debated until consensus could be reached. Of the four studies asking people with intellectual disabilities about their self-injury, Didden *et al.* (2008) and Harker-Longton and Fish (2002) scored nine, Brown and Beail (2009) scored eight and Duperouzel and Fish (2010) seven.

### Data extraction and synthesis

Data on self-reported reasons for self-injury were extracted into a separate findings document for analysis and synthesis. (See Table 4: Data Extraction). An iterative approach was taken, in which the findings were read and re-read to identify themes inductively; themes were driven by the findings of the studies rather than theory. The data set was coded systematically before collating into potential themes. The dominance of a theme was gauged according to the frequency across the data set as well as the weight given to it in individual studies. Quotes from participants were used to illustrate particular themes and in acknowledgement of the importance of the participants’ voice in this review. Some latent interpretation was applied—for instance, when the participant in Harker-Longton and Fish’s (2002) study was asked about her self-injury, she replied: ‘“it still hurts. Pain in there.” [Points to her heart]’ (p. 143). This was coded as an example of ‘difficulty articulating emotions’ as the participant appeared to be struggling to name her distress, instead referring to it in physical terms. Thus, this review is embedded in a constructionist paradigm in which meaning is socially produced and reproduced.

**Table 4:** Data extraction summary of the four studies asking people with learning disabilities about their own self-injury

**Author/year**	Title	Coun-try	Sample	Aims	Design and analysis	Key findings	Quality Assess-ment Score
Didden et al. (2008)	Individuals with Prader-Willi syndrome and their perceptions of skin-picking behaviour.	The Netherlands	Ten people with a confirmed diagnosis of PWS and with a mild to borderline range of Intellectual disability (ID). (Aged 29-54). All displayed chronic skin picking.	We aimed to explore what these individuals thought about this behaviour in general, which adverse consequences (if any) this behaviour has and what they thought about why they show this behaviour and what elicits it' (p. 124).	semi-structured interviews and thematic analysis	Findings: general picture—all individuals scratched and picked with their nail and had done so for a long time. Reasons: medical and physical—because of eczema or itchiness, but also 'this behaviour belongs to my Prader-Willi syndrome, it will not disappear' (p. 126); psychological—'nerves', 'being teased', 'brooding', loss and boredom. Prevention: eight participants felt there was no remedy. two felt distraction, reward and prevention might help. Own perception: guilt, anger, that it was 'filthy', and that they felt bad about doing it. They felt shame at the scars. A feeling that they needed to 'make amends' for it.	9
Author / year	Title	Coun-try	Sample	Aims	Design and analysis	Key findings	Quality Assess-ment Score
Brown and Beail (2009)	Self-harm among people with intellectual disabilities living in secure service provision: a qualitative exploration.	UK	Nine people with ID living in a secure service who had engaged in SH in the last three months	To explore the experiences and understanding of SH (self-harm) among people with mild ID (intellectual disabilities) living in secure accommodation; also to explore the experiences of interventions associated with SH.	Semi-structured interviews, using an IPA framework and analysis.	Themes: **interpersonal relationships:** ‘traumatic early experiences of loss and abuse hold meaning in relation’ to SH. Current relationships were also significant, with loss of control and frustration with others considered a trigger. **SH was also ‘protective’**—an alternative to intense feelings of anger or distress. **Emotional experience:** feelings of anger, hopelessness, guilt and shame preceded SH; catharsis and lack of pain during SH; guilt and regret following SH. **Managing SH:** internal control, self-talk or seeking help; external controls such as removing property or restraint. Overall, the need ‘to consider the emotional world of people with’ ID highlighted. Noted that there were some themes which fit with the functional analysis model, but that other themes ‘add depth to such a formulation’.	8
Author/year	Title	Coun-try	Sample	Aims	Design and analysis	Key findings	Quality Assess-ment Score
Harker-Long-ton and Fish (2002)	'Cutting doesn't make you die': one woman's views on the treatment of her self-injurious behaviour.	UK	One woman with ID	To explore the personal perspective of SIB from a person with mild ID	Single case study using IPA	Reasons/functions of SIB: Upset before self-injuring and happy afterwards, but then very down an hour after or so. Explained that there was hurt inside her. That she needed to punish herself ‘for being dirty’ (p. 143), and the need for people to see that she had hurt herself as a way to communicate her distress. ‘whatever I’m sad about it’s steam coming out. A rush of stuff, stuff inside’ (p. 143) —a sense of getting some release and relief from the internal things. ‘Release, frustration’ (p. 143). There is also a sense of asserting control and power in Catherine’s narrative that resonates with mainstream literature (p. 147). Catherine believes that she will develop a medical illness if she doesn’t self-injure. This highlights the very personal reasons people self-injure. There was an emphasis from Catherine of the importance of the relationships with the staff around her.	8
Author/year	Title	Coun-try	Sample	Aims	Design and analysis	Key findings	Quality Assess-ment Score
Duperouzel and Fish (2010)	'Hurting No-One Else’s Body but Your Own: People with Intellectual Disability Who Self Injure in a Forensic Service'	UK	Nine people with ID, four males, five females	‘To capture the meanings attributed to self-injury and the perceptions that people with mild/moderate intellectual disability who self-injure have of their care in a medium secure unit’ (p. 607).	In-depth, unstructured interviews and a phenomenological design and analysis	Themes: Coping: ‘relief from extreme emotional states’ and ‘getting your feelings out’. Feelings of guilt and shame following self-injury perpetuated the need to do it again. Habitual and necessary for coping. **Relationships with staff:** desire to talk about feelings, alongside desire to avoid higher levels of observation and supervision. Punitive responses from staff—anger and blame. Desire to be talked to in ‘personal terms’. **Special observation:** SH often led to more observation and removal of possessions, resulting in feeling punished and that staff resented them. **Thoughts for the future:** contributing to training for staff and being allowed to SH. Concludes: reasoning of participants was similar to reasoning from people without ID—SH ‘serving much the same functions … expression of emotional distress, … coping with intense feelings of anger, shame, guilt, powerlessness, abuse and blame’ (p. 612).	7

Abbreviations in Table: IPA—interpretative phenomenological analysis; ID = intellectual disabilities; SH = self-harm; SIB = self-injurious behaviour; PWS = Prader-Willi Syndrome.

The initial coding of themes by the primary researcher was shared with the research team. After discussion, a couple of themes, including guilt and shame, were dropped, as the data implied that they were better understood as interrelated experiences of self-injury, rather than a self-reported explanation for self-injury. This reflective process enabled the establishment of primary and secondary themes which better reflected the data set. The final analytic themes were reviewed in relation to the whole data set to ensure that they mapped the findings in a meaningful and illuminating manner and in relation to the research question.

## Results

**Figure 1.**
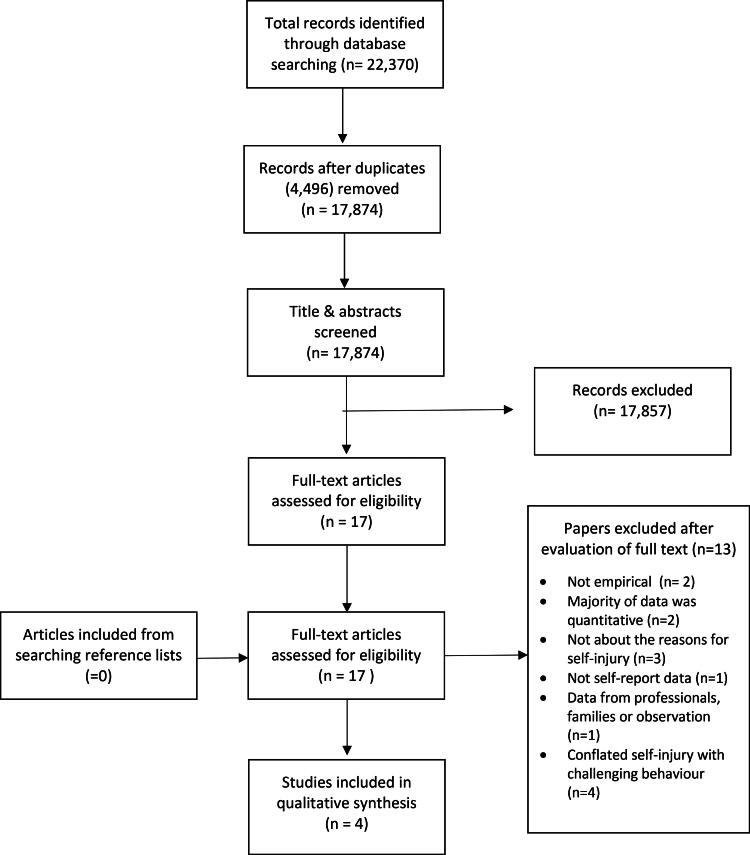
Flow chart of study identification. Adapted from PRISMA, 2020.^1^

Electronic database searches produced 22,370 articles. Duplicates were removed (4,496) leaving 17,874 articles. The primary researcher screened these by title and abstract against the inclusion/exclusion criteria, excluding 17,857 articles. The resultant 17 full text articles were screened against the inclusion/exclusion criteria: 13 were excluded, producing four articles representing four separate studies. (See Figure 1). The reference lists of the four articles were searched and no further relevant studies were identified.

The four studies were conducted between 2002-2010; no studies were found that had been published more recently than 2010. This is addressed in the discussion.

All four studies examined the experiences or perspectives of people with intellectual disabilities who self-injured. Three were conducted in secure or forensic services for people with intellectual disabilities in the UK (Brown and Beail 2009; Duperouzel and Fish 2010; Harker-Longton and Fish 2002). The fourth study (Didden *et al.* 2008), asked ten people with Prader-Willi Syndrome and intellectual disabilities in various community settings in the Netherlands about their skin-picking behaviour. Each of the studies utilised interviews as their primary data collection method; thus, it should be noted that the participants from the four studies all had some verbal communication, reflective of mild or moderate intellectual disabilities.

The researchers organised the self-reported explanations for self-injury into three primary themes: relief from overwhelming emotions; trauma and loss; and difficulty articulating emotions.

### Relief from overwhelming emotions

Regulating overwhelming emotion was a dominant theme in participants’ self-reported explanations for self-injury across all four studies. Duperouzel and Fish (2010) reported that ‘all the participants in this study felt that their self-injurious behaviour was helpful to them at times of emotional strain’ (p. 612). Similarly, Brown and Beail (2009) reported that:

self-harm was described as an intensely emotional experience and some described a relief of feelings…hopelessness, anger, frustration, guilt, regret and shame (p. 510).

The participant in Harker-Longton and Fish’s (2002) single case study described a build-up of emotions with a sense that self-injury enabled her to get some relief from these internal pressures. Didden *et al.* (2008) concluded that ‘most individuals in our study reported that an increased arousal level (e.g. nerves, being tense) often elicits skin picking’ (p. 128).

The language used by participants of included studies alluded to a build-up of emotional strain: ‘I just got more and more upset and agitated and I put my head through a window’ (Brown and Beail 2009, p. 508). Duperouzel and Fish (2010) reported that the phrase: ‘it helps me relieve pressure’ (p. 609) was commonly used to describe the function of participant’s self-injury:

I felt really bad, everything was getting on top of me, I couldn’t see a way out of it, and I did it [self-harmed], I didn’t feel any pain. It gets all my feelings out and you come back and you are happy (Duperouzel and Fish 2010, p. 609).

Similarly, participants from Brown and Beail’s (2009) study reported that self-injury ‘keeps me happy because then I know that I’m getting the frustration out’ and that ‘I felt calmer’ (p. 508) and Didden *et al.* (2008) reported that two participants said they felt ‘relieved after skin-picking’ (p. 127). ‘Relief from extreme emotional states that they were unable to cope with’ (Duperouzel and Fish 2010, p. 609) was a commonality across all studies and most participants.

### Trauma and loss

Adverse experiences were found as a factor relating to self-injury in three studies (all except Duperouzel and Fish 2010). Brown and Beail’s (2009) study reported this most emphatically: ‘this study suggests that traumatic past experiences are alive in the present and hold meaning in relation to self-harm’ (p. 510). Past relationships, particularly those involving abuse or loss, were a main theme:

events in the past… seemed unresolved…Most striking within this were participants’ experiences of sexual, physical or emotional abuse and loss, which they closely identified with self-harm (Brown and Beail 2009, p. 506).

Previous loss was reported as a factor in Didden *et al.*’s (2008) study, with one participant reflecting, ‘I have no father anymore…I often see my father standing in my bedroom, then I feel very nervous and stressed. Then I start picking at my skin’ (p. 126). Similarly, a participant in Brown and Beail’s (2009) study described the death of his friend, commenting, ‘that’s how it all started and that’s how I got into self-harming’ (p. 506). Catherine, from Harker-Longton and Fish’s (2002) study articulated a deep sense of loss and abandonment:

They leave me always…I feel rejected and lost, it always happens, they leave. [long pause] Everyone does (p. 145).

When asked what might help her with her self-injury, Catherine responded: ‘a psychologist. Talk about past things. [long pause]. That helps’ (p. 146). This was suggestive of previous adverse experiences. Brown and Beail (2009) found that their participants sometimes reported several adverse experiences:

this reflects the multiple traumas faced by many of this group which is also evident in some people suggesting more than one ‘reason’ for their self-harm as they sought to make sense of it (p. 506).

### Difficulty articulating emotions

Three of the four studies reported that their participants had difficulty articulating their emotions (all except Brown and Beail, 2009). Participants of the three other studies appeared to have some difficulty articulating their emotions, instead relying on physical or verbal expressions, and related this as one of the reasons for their self-injury, as is exemplified in this exchange from research by Harker-Longton and Fish:

[Interviewer] What do you think when you injure yourself? [Catherine] ‘It still hurts. Pain in there’. [Points to her heart] (Harker-Longton and Fish 2002, p. 143).

Self-injury was perceived to operate as ‘a form of communicating…distress’ (Harker-Longton and Fish 2002:147) or ‘a form of expression of emotional distress’ (Duperouzel and Fish 2010, p. 612). There was a desire to talk about self-injury, alongside a concern that they would not be able to express themselves adequately or that they would not be understood:

I self-harm and that’s all I’ve said…I don’t really go into that much detail. I feel scared. I don’t think they’d understand (Duperouzel and Fish 2010, p. 611).

Sometimes, for the participants in the included studies, it seemed that self-injury spoke for them in a way that they were unable to articulate through words. For example, when the interviewer asked a participant why she sometimes swallowed sharp and dangerous items, she responded: ‘Makes a point that I’m unhappy’ (Harker-Longton and Fish 2002, p. 143). Duperouzel and Fish (2010) found that some participants ‘were unable to verbalize or face up to these emotions and could not see a future without injuring themselves’ (p. 610).

## Discussion

### Key findings of studies about people with intellectual disabilities

In response to this review’s inquiry as to the explanations that people with intellectual disabilities give for their own self-injury, three primary themes were identified: relief from overwhelming emotions; trauma and loss; and difficulty articulating emotions. In addition to these main themes, some participants with Prader-Willi Syndrome in one study linked their self-injury with the diagnosis (Didden *et al.* 2008). Prader-Willi Syndrome, similarly to other syndromes such as Lesch-Nyhan, Cornelia de Lange and Fragile X, has an established etiological link to self-injury (Oliver and Richards, 2015). The other three studies did not report whether participants had any conditions in addition to having an intellectual disability.

The themes from the self-reported explanations for self-injury in these four studies echo the psychosocial reasons for self-injury that are well established in the wider literature (Kelada *et al.* 2018; Nock 2009). In order to make sense of this, we need to consider self-harm in people without intellectual disabilities. Through a similar systematic review process, we identified forty-four studies that asked people within the general population who self-harm the reasons for doing so^7^.

The findings relating to people within the general population were strikingly homogenous, despite a considerable breadth of theoretical lens, time frame and geography spanned. It seems that self-harm and the self-reported reasons for it have more similarities than differences ‘across cultural contexts’ (Tan *et al.* 2019, p. 16). The primary themes in people within the general population (affect regulation, (regulating both overwhelming emotions and numbness); adverse experiences; and emotional inarticulation) echo the primary themes found in the four studies asking people with intellectual disabilities about self-injury.

There were, however, some differences between the studies asking people with and without intellectual disabilities about their own self harm. Participants with intellectual disabilities did not mention regulating dissociation and numbness. Participants within the general population described self-hate, rumination and help-seeking as factors leading to self-harm; these were not reported in the four studies with people with intellectual disabilities. The 44 studies with participants within the general population had a different emphasis with regards to nameable emotions: anger, shame and guilt were more clearly articulated and comparatively more commonly described. These differences might be accounted for by the complexity of notions such as dissociation, guilt or rumination; it may be that those with intellectual disabilities asked about their self-injury were less able to articulate these more complex concerns.

### The disparity between people with intellectual disabilities and those without intellectual disabilities

The main disparities between the two sets of studies were in the quantity and time-frame of the studies. Our systematic review only found four studies asking people with intellectual disabilities directly about their own self-injury, and none of these were conducted in the last ten years. This was in contrast to 44 studies, of which 26 were published in the last ten years, asking people within the general population about their own self-harm: a disparity of almost 11:1. The body of research examining ‘self-injurious behaviour’ in people with intellectual disabilities is generally considered to be at least 50 years old (Murphy 1999; Symons *et al.* 2012). Whilst typical self-report studies are not possible or less reliable in those with more severe intellectual disabilities, it is still surprising that there have been none conducted at all with those with mild or moderate intellectual disabilities in the last ten years. Thus, the total absence of research asking people with intellectual disabilities about self-injury in the last 10 years requires some exploration. This is discussed in relation to inclusive research, ‘challenging behaviour’ and the biobehavioural model.

#### Inclusive research

For more than thirty years, proponents of the social model of disability have rejected the traditional view of research—that of specialist researchers doing research on subjects—and raised an expectation that research includes and involves people with disabilities (Oliver 1992; Oliver *et al.* 2012).^8^ This notion that, in research, there should be ‘nothing about us without us’ (Charlton 1998) has been—at least nominally—adopted by national bodies, including the UK government (Beresford 2013; Department of Health 2001). Inclusive research—which applies and extends emancipatory and participatory agendas to people with intellectual disabilities—has utility at heart (Nind and Vinha 2014). It seeks to ensure that, from policy to service delivery to culture, the needs and experiences of those that research concerns are being accurately understood (Woelders et al. 2015). With this in mind, the apparent absence over the last ten years of academic research that seeks the experiences of people with intellectual disabilities who self-injure is notable. Griffith *et al.* (2013) concur, reflecting towards the end of their integrative review:

This review found that there was a paucity of information in the original studies about service user experiences …[this] is somewhat surprising… Information about direct experience of service users is needed to inform intervention design and delivery, and behavioral interventionists ought to be gathering data from service users as part of an understanding of the social validity of the interventions (p. 484).

Collecting data about direct experience or self-report can be complex with participants with intellectual disabilities, as the ordinary tools such as direct interview, questionnaires or focus groups may need adjusting for some participants or may not be appropriate at all for others. However, there are good examples of inclusive research utilising augmented communication (Bradshaw *et al.* 2018; Pickering *et al.* 2015) and it is essential that research continues to utilise creative tools which facilitate the inclusion of people with intellectual disabilities in research (Nind and Vinha 2014).

#### Self-injury (‘self-injurious behaviour’) as ‘challenging behaviour’

All four papers in this systematic review were published more than ten years ago. More recent equivalent research has incorporated self-injury within the wider concept of ‘challenging behaviour’. Griffith *et al.* (2013) and Van den Bogaard *et al.* (2019) both report on the experiences of people who self-injure within their reviews of people’s experiences in relation to challenging behaviour. Van den Bogaard *et al.* (2019) include in their conceptualisation of challenging behaviour ‘aggression, SIB, stereotypic behavior, agitation, disruptive or destructive acts, withdrawn behavior, arson, and sexual misconduct’ (p. 128). There is no equivalent for this in research in people without intellectual disabilities. Research enquiring as to the reasons for self-harm is not situated within the wider discussion of why someone might be aggressive, set fire to something or sexually assault someone.

Self-injury does fit within the confines of the definition of challenging behaviour, but it is problematic (Minshawi *et al.* 2015). Challenging behaviour, by its very nomenclature, considers the interactional and proximal nature of a person’s response. Behaviour is the way in which we act and interact with those around us and/or the environment—the way in which people conduct themselves in response to situations or stimuli (Nock 2009). If self-injury is only seen through a behavioural lens, it is potentially reduced to a response to situations and stimuli. However, the findings of this review indicate that self-injury is not just a response to proximal factors, but can also be shaped by internal, distal factors such as emotional regulation difficulties which may be connected to experiences of trauma or loss. This suggests the importance of self-injury being considered in its own right as being a particular response in relation to a discrete set of aetiological factors.

#### The biobehavioural model

Research founded on biological and behavioural schools of thought have offered a long-term lead for research and practice concerning self-injury (Fahmy and Jones 1990). It has been argued that biobehavioural models ‘present a plausible account of how self-injury can be maintained [but] they cannot explain the origin of the behaviour’ (Fahmy and Jones 1990, p. 269). These concerns, well-articulated 30 years ago, persist: ‘…researchers have typically focused on the outcome of the behaviour rather than the behaviour itself’ (MacLean *et al.* 2020, p. 679).

Research with the general population has addressed this, in part, by asking those who self-harm directly (Lewis and Hasking 2019). In contrast, the research exploring ‘self-injurious behaviour’ has typically been dominated by drug trials, investigations into biological causations and behavioural interventions (Murphy 1999; Richards and Symons 2018); this is partly influenced by the complexities inherent in direct involvement of those with more severe intellectual disabilities, who are at greater risk for self-injury (Oliver and Richards, 2015).

The biological and behavioural schools of thought offer core components for understanding self-injury, including the efficacy of conducting functional behaviour analysis, and have positively influenced policy and practice for how individuals should be supported (NICE, 2013; Oliver and Richards, 2015). Behavioural research has made great strides forward in how we support people with intellectual disabilities who self-injure: it asserts that self-injury is a communication about something, and needs addressing individually and thoughtfully as a functional, adaptive response. However, there have also been warnings about the risks of functional behaviour analysis being too restricted:

functional analysis can lead to a narrow perspective such that practitioners of behaviour analysis may come to believe that all behaviour is a function of its consequences, thus leading them to ignore or discount other possible causative factors…[such as] lack of control, a loss of a love object and a disturbance in pain perception (Fahmy and Jones 1990, p. 271).

Overall, this has meant that self-harm, which amongst those without intellectual disabilities is widely taken to indicate emotional difficulties, is usually considered through the biobehavioural research within settings for people with intellectual disabilities; this can mean that the role of emotional difficulties related to wider adverse experiences, are not considered adequately (Lovell, 2008). Consequently, ‘there is a tendency to ascribe the emotional difficulties experienced by the individual to the disability rather than to emotional state or needs’ (Hollins and Sinason 2000, p. 32).

The principles behind functional analysis are not disputed—self-harm can often serve a function for an individual. However, the focus on antecedents may limit possible causes of self-harm to proximal factors only: ‘what happens before, during and after the behaviour’ (BILD 2016:2). In contrast, for a person without intellectual disabilities, distal factors—loss, bullying, trauma, familial tension, abuse—are considered as possible factors from the outset (NICE 2013).

According to the limited evidence available, reasons for self-injury given by those with mild and moderate intellectual disabilities include distal factors, such as previous experiences of trauma and loss. As a person’s intellectual disability increases in severity, consideration of distal factors becomes more complex, as the individual is less likely to be able to share the nature of their wider experiences or be able to necessarily link them to their self-injury. However, it seems important that the wider experiences and emotional lives of people with intellectual disabilities are considered, wherever possible, alongside proximal, behavioural factors. Whilst challenging, this is not out of the question, where good multidisciplinary practice is implemented (Heslop and Macauley 2009; Lovell 2008).

### Recommendations for further research

Despite the proliferation of research into self-harm in people within the general population over the last twenty years (Cipriano *et al.*, 2017), studies continue to conclude by raising concerns about the paucity of applicable research, policy initiatives and training (Borschmann and Kinner 2019). Although further research asking people within the general population about self-harm is urgently needed (Lewis and Hasking 2019), there is an even greater dearth of recent research about the views of people with intellectual disabilities about their reasons for self-injury. This has created a situation in which their lived experiences and self-reported reasons for self-injury are not adequately reflected in policy and practice.

Research that seeks the views of people from the whole spectrum of intellectual disabilities who self-injure is also required. Seeking self-report contributions from people with different communication needs or more significant intellectual disabilities poses significant challenges around consent, capacity and methods; however, examples of creative methods are burgeoning (Runswick-Cole *et al.* 2018), including adapted ethnographic methodologies (Klotz 2004). These challenges can be overcome, so that ‘all people with intellectual disabilities are … accepted and engaged with as inherently social and cultural beings’ (Klotz 2004, p. 101). It is also important that the views of people with intellectual disabilities from a range of settings, including the community, are asked about their experiences of self-injury.

The third theme was ‘difficulty articulating emotions’. Difficulty in recognising and articulating emotions, sometimes called alexithymia, has been highlighted as a concern for people with intellectual disabilities (Davies *et al.* 2015). This is a wider difficulty associated with the communication deficits associated with intellectual disabilities. However, there is some interest in the relationship between alexithymia and challenging behaviour (Davies 2013; Mellor and Dagnan 2005), which warrants further exploration in relation to self-injury.

Wider research into the experiences of people with intellectual disabilities who self-injure appears to have been encapsulated into research about ‘challenging behaviour’. However, the literature which adopts this method, reports separate features and associations for self-injury. Future research should reconsider the appropriateness of only enquiring about people with intellectual disabilities who self-injure within the confines of ‘challenging behaviour’.

Finally, Cognitive Behaviour Therapy (CBT) and Dialectical Behaviour Therapy (DBT) are recommended treatments for people in the general population who self-harm (NICE, 2013). Initial work adapting these therapies for people with intellectual disabilities is promising (Crossland, Hewitt and Walden 2017; Nair, Woodrow and Hare, 2016), but more research exploring how best to adapt and apply these therapies with people with a range of intellectual disabilities is still needed.

### Strengths and limitations of the included studies and the review process

The systematic review ensured a rigorous approach through reflecting on the PRISMA (2020) guidelines for reporting a systematic review, and utilising the CASP (2018) checklist for rigorous quality assessment of the studies. The screening and selection process and the quality assessment were double checked by a second reviewer to ensure rigour; the themes and findings were also discussed.

There are, however, some limitations to the findings of this study. The first is that the published evidence for people with intellectual disabilities who self-injure is not contemporaneous, dating back more than a decade. Understanding of self-injury has developed over the last ten years, and yet there have been no studies asking people with intellectual disabilities about their own self-injury. This is significant gap in the evidence available.

Secondly, all four studies included participants with mild to moderate intellectual disabilities. Thus, the views of people with a range of intellectual disabilities have not been reported. There is an increasing acknowledgement of 'nonverbal, bodily, sensory and emotional forms of communication and dialogical competence’ (Gjermestad *et al.* 2022:2), as well as the neurodiversity present for people with severe and profound intellectual disabilities in understanding and communicating about the world. These ‘voices’ need to be included in research, using creative and imaginative methodological and theoretical means (Klotz 2004). Alongside this, it is acknowledged that direct self-report data, which implies verbal contribution from participants, is not attainable in a straightforward manner from those with more severe intellectual disabilities. Methods for gathering nonverbal self-report are inherently interpretative, requiring explicit creation of meaning in a more deliberate and comprehensive manner than verbal self-report data implies (Gjermestad *et al.* 2022).

Thirdly, three of the four studies of people with intellectual disabilities were conducted in secure or forensic settings in the UK; only Didden *et al.*’s (2008) study was outside the UK. Institutional settings are recognised as a factor for self-injury for people with (Fish 2018) and within the general population (Mangnall and Yurkovich 2010); institutionalisation erodes an individual’s sense of agency, which can lead to utilising self-injury as a form of regaining a sense of control of the body (Fish 2018). The higher proportion of participants in the studies from secure settings may have impacted the findings, increasing the likelihood that the sample represents individuals with dual diagnosis of intellectual disabilities and mental illnesses.

Fourthly, the small sample size of participants in the studies included in the systematic review needs to be acknowledged; Harker-Longton and Fish’s (2002) study had a single participant only, which may have overrepresented their experience. The studies all interviewed a mix of male and female participants, with the exception of Harker-Longton and Fish (2002) which had one female participant.

Fifthly, the results of the studies were analysed rather than the raw data.

Lastly, the qualitative nature of the included studies and the narrative nature of the review means that author bias must be considered. The primary researcher acknowledges that prior professional experiences of supporting young people with intellectual disabilities who self-injure, and current research seeking the views of those with severe intellectual disabilities who self-injure about their emotional experiences, may have influenced the review. The researcher also acknowledges a political bias towards emancipatory research involving people with intellectual disabilities.

## Conclusion

This systematic review addressed the research question: ‘what reasons do people with intellectual disabilities (who are able to self-report) give for their own self-injury?’ It found that people with mild and moderate intellectual disabilities gave very similar explanations for their self-injury as participants from the general population. These explanations were that self-injury provided some relief from overwhelming emotions, which were linked to previous experiences of trauma and loss; difficulty in articulating these emotions was also connected to self-injury.

People with intellectual disabilities are not a homogenous group, and the self-report data from those that can give it (typically those with mild and moderate intellectual disabilities) cannot be transferred to those with more severe intellectual disabilities who are not easily able to participate in ordinary self-report studies. However, the findings resonate with other research arguing that the well-established psychosocial understanding about why people self-harm (Klonsky 2009; Nock 2009) should not be discounted for people with intellectual disabilities (Heslop and Macauley 2009; Lovell 2008; Van den Bogaard *et al.* 2019).

Some proponents of the biobehavioural model have expressed concerns that efforts expended on identifying and treating psychosocial factors might mean that behavioural or biological functions are overlooked, or even that interventions aimed at mitigating emotional distress might reinforce a learned behaviour (Oliver and Richards 2015). Advocates for a psychosocial approach argue that, if an individual’s self-injury is a way to communicate distress, particularly in relation to adverse life experiences, then ‘continually ignoring the “message” in self-harm is to further traumatize individuals’ (Jones *et al.* 2004, p. 487).

However, behavioural interventions implemented with people who self-harm in the general population sit very comfortably alongside psychosocial theory and practice (Klonsky 2009). Similarly, for people with intellectual disabilities, the psychosocial model, which considers emotional regulation, traumatic experiences including loss, and difficulties articulating emotions can intersect and overlap with the biobehavioural model. For instance, the report from Harker-Longton and Fish’s (2002) study, in which the participant explains that she swallows sharp and dangerous items as it ‘makes a point that I’m unhappy’ (p. 143), can simultaneously be interpreted as a struggle to articulate overwhelming emotion (psychosocial model), or a bid for much-needed attention (behavioural model). Multidisciplinary approaches, which reflect the intersection of all the models and approaches, are needed for people with a range of intellectual disabilities who self-injure (Heslop and Macauley 2009; Minshawi *et al.* 2015).

Finally, for people with intellectual disabilities, self-injury should be considered a discrete concern rather than another form of challenging behaviour. Traditional behavioural charts often ask about the ‘trigger’ or ‘antecedent’ for self-injury; this only considers proximal factors. Distal factors should also be considered, particularly reflecting on the higher risk that people with intellectual disabilities have to experiencing discrimination, stigma (Marriott *et al.* 2020) and abuse (Miller and Brown 2014), as well as considering any recent losses (Dowling *et al.* 2006). This is particularly pertinent in light of the higher risks apparent for people with intellectual disabilities to adverse experiences and multiple vulnerabilities (Brown and Beail 2009; Minshawi *et al.* 2015).

## Notes

1 The use of terms is far from consistent within the literature. This is well-illustrated by the variety of terms used in the four research studies presented in this systematic review, which use the terms self-harm, self-injury and self-injurious behaviour with some variability. The term self-injury is adopted in this paper in line with the NICE guidance (2015).

2 The National Institute for Clinical Excellence provides evidence-based recommendations for UK health and social care.

3 The term ‘Learning Disability’ is commonly used in the UK; it is an equivalent term to ‘Intellectual Disabilities’.

4 The term ‘general population’ is used here to denote research conducted in settings which do not exclusively support people with intellectual disabilities. However, much research undertaken with people who self-harm is conducted with groups of people who are high-risk for self-harm, such as adolescents, prison populations and those accessing a variety of mental health services (Cipriano, Cella and Cotrufo 2017).

5 Studies classed as being with people from the ‘general population’ were studies that did not report or mention having participants with intellectual disabilities. However, it is acknowledged that they may have included participants who fit the criteria for having intellectual disabilities, whether undiagnosed or unreported.

6 Challenging behaviour is recognised as an inherently problematic term: it labels potentially valid emotional expression as ‘behaviour’ – and as a behaviour that challenges others - problematizing it in terms of the inconvenience to others rather than placing the person’s needs at the centre; this is addressed further in the discussion.

7 None of the studies reported that participants had intellectual disabilities, though it is possible that some participants may have had mild or undiagnosed intellectual disabilities.

8 The social model of disability, whilst recognising the potential restrictions of mental or physical impairment, states that it is society that disables by excluding those with impairments (Oliver, 1992).
